# Rehabilitation Outcomes of Cortical Blindness and Characteristics Secondary to Cardiac Arrest: A Review

**DOI:** 10.7759/cureus.32927

**Published:** 2022-12-25

**Authors:** Deklerk A Ngankam, Kelley Crozier, Anh-Thu Vu

**Affiliations:** 1 Physical Medicine and Rehabilitation, University of Pittsburgh Medical Center, Pittsburgh, USA; 2 Physical Medicine and Rehabilitation, Reading Hospital Rehabilitation at Wyomissing, Reading, USA; 3 Neurology, Drexel University College of Medicine, Philadelphia, USA

**Keywords:** post-cardiac arrest, behavioral and psychiatric symptom, cognitive dysfunctions, physical medicine and rehabilitation, cortical blindness, visual field defect, rehab, pca infarction, occipital lobe infarct, vision rehabilitation

## Abstract

We reviewed the published literature on rehabilitation outcomes in patients with cortical blindness (CB) and highlighted the characteristic features and prognosis of CB due to cardiac arrest. The studies excluded were those involving the pediatric population (<age 16), written in a language other than English, and studies with no mention of outcomes. The literature search was done by PubMed and EBSCOhost databases from the oldest available literature through November 2019. Due to the scarcity of published literature and a qualitative description of outcomes, a narrative review of the literature was deemed appropriate. Seven case reports and one retrospective cohort study met the inclusion criteria. Cognitive and visual impairments were significant barriers to rehabilitation in CB. Improvement of visual deficits occurred within one to two months. Those with complete blindness, cognitive impairments, and a delay in resuscitation were more likely to have poorer functional outcomes in the performance of activities of daily living and were less likely to be discharged home. This is the most comprehensive review of published literature to focus on the function of patients with cortical blindness. The limitations include the small number of published literature and the qualitative approach utilized. Despite the limitations, the findings of this review can inform future studies that would investigate the most efficient and comprehensive methods of CB rehabilitation.

## Introduction and background

Cortical blindness (CB) is a type of vision loss resulting from bilateral occlusion or flow reduction within the posterior cerebral arteries (PCAs). Specifically, the blood supply to the geniculocalcarine visual pathways of the occipital lobes is compromised, leading to bilateral vision loss [1]. PCA territory infarcts account for 5%-10% of strokes [2]. Numerous causes of CB have been reported, including ischemic stroke, cardiac arrest, vasospasm of the posterior cerebral arteries, meningitis, and angioedema [3-10]. In a study of 25 patients, spontaneous ischemic stroke was the most common cause of CB, followed by cardiac surgery and cerebral angiography [8]. Complete cortical blindness is less common than partial cortical blindness or homonymous hemianopsia (from unilateral damage to the visual pathway), and some visual functions, such as light or motion perception, are usually preserved. Central vision is preserved (also known as "macula sparing") due to dual blood flow to the posterior aspect of the calcarine fissure of the occipital cortex supplied by PCAs and middle cerebral arteries. In patients with CB secondary to stroke, the onset of CB varied, from sudden blindness to gradual worsening of vision over minutes to hours to vision loss that developed several days later [1]. Patients may simultaneously experience confusion, disorientation, visual hallucinations, and anosognosia (denial of blindness or lack of awareness of visual deficits) [11-14]. Anton syndrome is a clinical syndrome seen in some patients with cortical blindness and includes the additional symptoms of anosognosia and confabulation. The recognition of CB is delayed and is likely due to a combination of the variation of CB onset among patients, patient and medical factors such as patient denial or unawareness of blindness, and subtle findings on brain imaging that may be overlooked or lead to alternative diagnoses [15].

While numerous studies focus on vision rehabilitation methods in patients with visual field deficits and cortical blindness [16-17], studies that focus on functional outcomes, e.g., activities of daily living in patients with cortical blindness are scarce. One study investigated the rehabilitation outcomes of patients with PCA stroke but did not include patients with cortical blindness. Cardiac arrest is one cause of cortical blindness due to cerebral hypoperfusion from systemic hypotension [6], but reports on prognosis with this etiology are also scarce. Aldrich et al. reported that patients with CB secondary to spontaneous stroke have a worse visual prognosis than other causes of CB. However, their study had only one case of CB caused by cardiac arrest out of the other eight cases of CB caused by non-spontaneous stroke. A small cohort of patients with cortical blindness secondary to cardiac arrest has been previously described, but it was a single-institution review, and the focus was on imaging abnormalities of the occipital lobes [15].

The purpose of this paper is to review the published literature on rehabilitation outcomes in patients with cortical blindness. Ischemia secondary to cardiac arrest is more likely to cause cortical blindness due to bilateral deficits because of hypoperfusion. Given this unique pathophysiology, we decided to look at the characteristics and prognosis of cortical blindness due to cardiac arrest. The authors hope that this will enhance understanding of the current knowledge of CB secondary to cardiac arrest and stroke and aid in the counseling of patients and their families on the impact that CB has on their functional outcomes.

This article was previously presented as a meeting abstract at the 2022 Association of Academic Physiatrists Annual Meeting on May 27, 2022.

## Review

Methods

A literature search was performed using the PubMed MeSH (Medical Subject Headings) and EBSCOhost databases. The following terms were used in the search: "cortical blindness [MeSH]," "rehabilitation [MeSH]" or "function," "cortical blindness [MeSH]" and "heart arrest [MeSH]," and "outcome" or "prognosis." The references to the papers were then reviewed to find additional literature on the topics of interest. The search timeframe was from the oldest available literature on PubMed through November 2019. The studies excluded were those involving the pediatric population (<age 16), those written in a language other than English, and studies with no mention of outcomes. Functional rehabilitation outcome was determined by performance on activities of daily living (ADLs), mobility or ambulation, or the Functional Independent Measure (FIM), a validated rehabilitation outcome tool used in assessing 13 motor and five cognitive tasks that is reliable in measuring functional ability [17]. Due to the scarcity of published literature on these subjects and a qualitative description of patient outcomes, a narrative review of the literature was deemed appropriate.

Results

Only three studies were found on the functional outcome of rehabilitation in patients with cortical blindness: two case reports [7, 18] and one retrospective cohort study [19] consisting of nine patients in total (Table 1). Patients with complete cortical blindness were more likely to have cognitive and behavioral signs (confusion, insomnia, memory problems, restlessness) and had poorer rehabilitation outcomes. They showed minimal to no improvement in performing activities of daily living (ADLs) and were more likely to be discharged to nursing facilities. The patient with bilateral occipital or thalamic infarction was an exception; he initially responded poorly to rehabilitation efforts but eventually became capable of interacting with his environment, which coincided with an improvement in cognitive, behavioral, and visual functions (Table 1).

**Table 1:** Summary of patient characteristics and rehabilitation outcomes N: the number of patients and their corresponding etiologies of CB;  FIM: Functional Independence Measure; ADL: activities of daily living; CTE: cognitive therapeutic exercises [18]

Etiology (N)	Complete or partial blindness	Cognitive/behavioral signs	Rehabilitation outcome
Bilateral ischemic occipital/thalamic infarction (1)	Complete	Confusion, restlessness	After 12 weeks, the patient was able to do basic self-care.
Bilateral ischemic occipital infarction (1)	Complete	Poor memory and concentration	Improvement in FIM (+15): transferring, dressing, and toileting; ambulated 150 feet with minimal assistance and a wheeled walker
Bilateral occipital hemorrhage (2)	Complete	Confusion, agitation, aggression, akathisia	No improvement
Bilateral occipital infarction secondary to cardiac arrest (1)	Complete	Related to visual perception and imagery, including difficulty in perceiving, constructing, processing, and remembering visual information.	Independent for basic ADL: ambulated 150 feet and negotiated stairs; needed less assistance with arm movement and was independent (after eight months of CTE).
Bilateral ischemic occipital infarction (2)	Partial	Memory impairment	Able to do self-care and leisure activities
Unilateral occipital infarction (2)	Partial	Memory impairment	Able to do self-care and leisure activities

In two of the four patients with cognitive and behavioral signs, occupational therapy was discontinued because of worsened confusion. Confusion interfered with learning compensatory mechanisms. Melatonin was used to control disturbed sleep patterns, and antiepileptics were used to control episodic aggression, with inadequate control achieved in some cases. In comparison, patients with partial blindness and fewer cognitive or behavioral signs showed excellent responses to rehabilitation (described as using environmental adaptations, e.g., illumination, magnifying lenses, etc., and strategies to cope with memory impairment using dictaphones). They were more likely to be discharged to their homes and families. One patient with complete blindness from bilateral occipital ischemic infarction gained 15 points in the Functional Independence Measure (Table 1). He showed improvement in insight and ADLs and ambulated 150 feet with minimal assistance by using a gait belt and fixed-wheel walker. His favorable functional outcome despite having complete blindness at the onset can be explained by the brain MRI at presentation: it showed a chronic left posterior cerebral artery (PCA) infarct and an acute right PCA infarct, in contrast to those with complete blindness and acute bilateral PCA infarcts. Still, one could theorize that the results show that patients with partial blindness and fewer cognitive or behavioral signs can have better functional outcomes because of an increased ability to participate in therapy.

Six case reports on cortical blindness secondary to cardiac arrests were found (Table 2). Parmar and Trobe previously reported two patients (cases three and four) [14]. Case three was previously reported by Aldrich et al. [1]. The recognition of cortical blindness secondary to cardiac arrest was delayed in all cases. The number of days that elapsed after blindness was recognized was between five and nine. However, in some cases, recognition of CB took longer: blindness in case four was not recognized until after she awoke from a coma on day 20. Another patient, case six, was sedated as part of a cooling protocol (to decrease cerebral edema) [20] and could only communicate his blindness on day 21 (even though a brain MRI on day five was indicative of ischemia of the mesial occipital lobes).

**Table 2:** Case reports of six patients with cortical blindness secondary to cardiac arrest NR: not reported; *The number of days elapsed after which blindness was recognized

Case	Authors	Age/sex	Resuscitation Time (min)	Blindness (days elapsed)*	Clinical presentation	Imaging	Outcome
1	Juan et al. [21]	22/F	NR	NR	Low visual acuity (“count fingers”), anosognosia, left side hemi-asomatognosia, visual agnosia, left hemiparesis and hemi-hypoesthesia	Cortical hyperintensity in both occipital lobes, precentral, corpus callosum splenius	Improvement of visual acuity during hospital stay of unknown duration.
2	Hoyt, Walsh [22]	39/F	3-5	9	Anosognosia, the inability to recognize small hand lights in all visual fields or perceive hand motions; spatial disorientation	NR	Slow return of vision between one and two months
3	Aldrich et al. [1]	26/M	NR	NR	Light perception only bilaterally, spastic dysarthria, and spastic quadriparesis.	Bilateral occipital abnormalities	No improvement one year later
4	De Patre et al. [18]	48/F	10	20	Inability to direct or maintain gaze to objects or to follow a light; tetraplegia; dysarthria.	Hypoattenuation with loss of gray-white boundary in bilateral occipital lobes	Poorly distinguished shapes and colors at 20 months after cardiac arrest
5	Margolin et al. [5]	16/M	5	5	Low visual acuity (hand movements in both eyes)	Subtle occipital gyri enhancement indicating a subacute ischemic lesion, atrophy of the medial occipital lobes	At three months, the vision was 20/20, with severely constricted visual fields; at one year, the visual acuity was 20/15, with slightly improved visual fields.
6	Kamyar, Trobe [20]	28/M	10	21	Only hand movement vision in both eyes	Hyperintense signal and loss of gray-white distinction in mesial occipital regions bilaterally.	Within one to two months, visual acuity improved to 20/200 in both eyes and bilateral homonymous hemianopia with macular sparing; > two months: 20/30 bilaterally, no change in visual fields.

Generally, vision improvement was noted between one and two months. Patients showed improvement in visual acuity within a few months. The visual field defects improved slightly but persisted for up to one year in case five. Complete recovery of vision was not observed or mentioned. Two patients (cases three and four) showed no improvement for up to one year. However, they had the worse vision at the onset. Case three could only recognize light, and case four could not perceive light or motion, which suggests an inverse relationship between the severity of the visual deficit at onset and the pace of recovery.

Discussion

The results from the review of published literature on the functional outcomes of rehabilitation of patients with CB suggest that patients with complete cortical blindness were more likely to have neurocognitive and behavioral symptoms, as well as poorer functional outcomes, compared to those with only partial blindness. In this study, 55% of the cases with bilateral infarcts or hemorrhages of the occipital lobes experienced neurocognitive and behavioral symptoms. We theorize that the poorer functional outcome of complete blindness results from the severity of the insult combined with the anatomical distribution of the PCA, as previously argued by Brandt et al. [4].

The PCA supplies the thalamic nuclei, limbic system, posterior limb of the internal capsule, cerebral peduncles, mesencephalon, and parts of the parietal, temporal, and occipital cortices [23]. While the most common cause of CB is ischemic stroke, cardiac arrest can affect a wide area of the PCA territory due to hypoperfusion. Cerebral hypoxia, lasting greater than three minutes and ten seconds, leads to permanent changes such as necrosis and swelling of regions of the brain (visual and motor areas, basal ganglia, deeper cortical layers) that are most sensitive to hypoxia due to their greater need for oxygen supply [2]. A compromise of adequate blood flow to the PCAs will lead to not only the development of blindness but also neurocognitive and behavioral symptoms because of the compromise of subcortical regions of the brain that control cognition and behavior [19]. Furthermore, patients who suffer a significant delay in cardiac resuscitation are more likely to have neurocognitive and behavioral symptoms and need more assistance with ADLs, low levels of satisfaction, and difficulty navigating environments, as observed in this review.

Interestingly, unlike the patients in Gaber who experienced confusion and agitation associated with CB, the cognitive impairments experienced by Case 4 were related to visual perception and imagery (e.g., difficulty perceiving, constructing, processing, and remembering visual information)^.^ This suggests that, in addition to neurocognitive and behavioral symptoms, the inability to navigate their environment could contribute to poorer functional outcomes.

There is a linkage between CB and the need for high assistance with ADLs, low levels of satisfaction, and difficulty navigating environments [15, 23]. The patient with bilateral occipital and thalamic infarction (Table 1), who initially responded poorly to rehabilitation efforts, showed improvement in function simultaneously with improvements in visual abilities. The association between visual impairments and poor functional ability in ADLs has been established [24]. It has led to the proposal of various vision therapy methods to address visual deficits in stroke patients. According to Melnick et al. [3], they fall under three classes: restitution therapies, which aim to restore visual deficits; compensation therapies, which rely on eye saccades to see visual information that would otherwise not be seen in the blind fields; and lastly, substitution therapies, which use optical devices or prisms to capture visual stimuli from the blind fields and relay them to intact parts of the visual field. There is insufficient evidence of the benefits of these methods on the outcomes of functional activities of daily living [25]. None are widely accepted and clinically validated.

To further highlight the interplay between visual abilities and functional outcomes of rehabilitation, De Patre et al. reported on the trial of the Cognitive Therapeutic Exercises (CTEs) rehabilitation approach on a cardiac arrest patient (case four, Table 2) who was dependent on ADLs and poorly differentiated shapes and colors 20 months after the cardiac arrest [18]. CTEs involved solving cognitive problems, correlating sensory and visual information, and moving limbs to reach, grasp, and manipulate objects. The goal was to increase ADL independence by increasing visual perception and imagery of two- and three-dimensional objects encountered in day-to-day activities. After eight months, visual and motor functions improved (namely, visual fields, ambulation, ADL independence, object recognition, and upper limb movements). After the rehabilitation approach, positron emission tomography (PET) images demonstrated an increase in focal glucose metabolic activity bilaterally in the occipital lobes. The authors suggest that the enhanced visual recovery following CTEs was due to the simultaneous visual and sensory stimuli, which produced increased metabolic activity in bilateral dorsal premotor areas and angular gyri (sites associated with shape and length discrimination tasks). They suggest juxtaposing visual and sensory stimuli in a rehabilitation program to reach a higher potential for visual and sensorimotor recovery. Paired with studies that have demonstrated a decrease in the blind field when one vision therapy intervention is combined with another [25-27], it is essential to consider an integrated approach in the therapy of visual defects or blindness. While it is unlikely that there will be complete recovery, therapy should be independent and focused on the patient’s goals and interests; e.g., if the patient is an avid reader, compensatory techniques can be tried, as there is some evidence that compensatory scanning training is more beneficial than a placebo at improving reading speed [25].

While the optimization of vision therapy methods can enable people with CB to engage with their environments, physiatrists can enhance participation in rehabilitation by considering pharmacologic agents such as lithium, valproic acid, carbamazepine, neuroleptics, and clonidine, which were found to be efficacious in treating irritability, aggression, and restlessness in post-stroke mania [28]. However, these treatments have not been examined in placebo-controlled, double-blind trials.

The conclusions drawn in this examination of the literature are limited due to several factors: (1) the small number of published literature found and included due to the scarcity of literature on the subjects of interest; (2) the qualitative approach utilized was necessary due to the inconsistency of outcome measures used; (3) the lack of additional pieces of data in some studies that would help determine relationships, such as the duration of cardiac arrest; and (4) the inconsistent reporting of long-term follow-up of the patients. Despite the limitations, the findings of this review can inform future studies that would aim to investigate the most efficient and comprehensive methods of CB rehabilitation. Future studies may want to consider the simultaneous assessment of visual impairment and neurocognitive and behavioral symptoms and their impact on functional outcomes.

## Conclusions

Patients with severe CB are more likely to have neurocognitive and behavioral impairments, which we suggest is directly proportional to the severity of the anatomical insult (extensive damage to the PCA territory extends into the subcortical regions of the brain). These patients were also more likely to have a poor response to rehabilitation efforts and, thus, poor functional outcomes from rehabilitation and disposition. We believe that these findings emphasize the role of multidisciplinary rehabilitation involving occupational therapists trained in vision therapy. Collaboration between physiatrists and neuropsychologists is essential to selecting evidence-based pharmacologic agents to manage neurocognitive and behavioral symptoms and facilitate patient participation during acute rehabilitation.
